# TAFM: A Recommendation Algorithm Based on Text-Attention Factorization Mechanism

**DOI:** 10.1155/2022/1775496

**Published:** 2022-08-29

**Authors:** Xianrong Zhang, Ran Li, Simin Wang, Xintong Li, Zhe Sun

**Affiliations:** ^1^Cyberspace Institute of Advanced Technology (CIAT), Guangzhou University, Guangzhou 510006, China; ^2^Academy of Digital Human Technology, Shenzhen Tianyuan Dic Information Technology Co., Ltd, Shenzhen 518057, China; ^3^Shenyang Institute of Computing Technology, University of Chinese Academy of Sciences, Shenyang 110168, China

## Abstract

The click-through rate (CTR) prediction task is used to estimate the probabilities of users clicking on recommended items, which are extremely important in recommender systems. Recently, the deep factorization machine (DeepFM) algorithm was proposed. The DeepFM algorithm incorporates a factorization machine (FM) to learn not only low-order features but also the interactions of higher-order features. However, DeepFM lacks user diversity representations and does not consider the text. In view of this, we propose a text-attention FM (TAFM) based on the DeepFM algorithm. First, the attention mechanism in the TAFM algorithm is used to address the diverse representations of users and goods and to mine the features that are most interesting to users. Second, the TAFM model can fully learn text features through its text component, text attention component, and N-gram text feature extraction component, which can fully explore potential user preferences and the diversity among user interests. In addition, the convolutional autoencoder in the TAFM can learn some higher-level features, and the higher-order feature mining process is more comprehensive. On the public dataset, the better performing models in the existing models are deep cross network (DCN), DeepFM, and product-based neural network (PNN), respectively, and the AUC score metrics of these models hover between 0.698 and 0.699. The AUC score of our design model is 0.730, which is at least 3% higher than that of the existing models. The accuracy metric of our model is at least 0.1 percentage points higher than that of existing models.

## 1. Introduction

Currently, the amount of information on the Internet is exploding [1], and it is becoming increasingly expensive for people to find information of interest. Recommendation systems can effectively alleviate the problem of information overload [2], and it is generally divided into two phases: recall and ranking. The ranking phases often need to be converted into a task of predicting the click-through rates (CTRs) of items.

The CTR prediction task, predicts the probability of a user clicking on a recommended item. Feature engineering and the automation of feature combinations are the main advancement directions of CTR prediction technology. Traditional support vector machines (SVMs) [3], Bayesian models [4], and logistic regression models [5] perform well in data-rich scenarios but require manual feature engineering and feature combination. Zhang et al. [6] and Xu et al. [7] used factorization machine models (FMMs) to automatically fit the interactions between features. This type of model is a matrix decomposition-based machine learning algorithm proposed by Rendle [8]; it combines the advantages of SVMs and factorization models in terms of linear complexity and generalizability. Then, it combines the integration ideas of bagging and boosting to conduct integrated learning for dealing with high-dimensional sparse problems, but a model based on an FM cannot learn deep feature interactions. Yu et al. [9] proposed utilizing the deep factorization machine (DeepFM) model to achieve personalized social ad recommendations. Wu et al. [10] proposed utilizing the deep cross network (DCN) model to better classify products in financial product malls. All these models have achieved good results in the recommendation field, but they cannot access the diversity of user interests at a deeper level.

Therefore, this paper proposes the text-attention FM (TAFM) algorithm, which can solve the problem of user interest diversity and fully exploit higher-order feature interactions by mining text features, designing attention mechanisms, and employing convolutional self-encoders. The specific contributions are as follows:

1. The TAFM model mines text features in a deep neural network (DNN) part. We design a DNN layer and an intent mining layer to form a deep layer. The intent mining layer includes a text component, a text attention component, and an N-gram text feature extraction component. The text component performs text feature extraction, and the long short-term memory (LSTM) [11] contained in this component solves the long-time dependence of text. Compared with RNN, LSTM effectively prevents gradient explosion and gradient dispersion problems via the introduction of gating units. The text attention component is used to mine the key information in the input text. This component is designed with bidirectional LSTM (BiLSTM)-Attention to extracting key information from the given text, and it uses this BiLSTM and attention mechanism to capture the most important semantic information contained in the sentences [12]. The attention mechanism automatically focuses on the words that have decisive impacts on the classification process and captures the most important semantic information in the sentences without utilizing additional knowledge and natural language processing (NLP) systems. The N-gram text feature extraction component is used to mine the N-gram features [13] in the text by designing CNN [14] that forms one-dimensional convolution representations. Multiple components can further mine the invisible textual intent features from the user's comment data.
2. In the embedding part of traditional deep learning prediction models, only simply designed embeddings layers are used for the features; they transform high-dimensional sparse features into low-dimensional dense features as model inputs. However, directly passing high-dimensional data into an embedding layer leads to excess parameters, impacting the effectiveness of the model. In this paper, we adopt the format of an FM based on domain knowledge and introduce the concept of fields. Second, in the embedding layer, this paper also designs a convolutional self-encoder, which can retain more information in the high-dimensional space and can tap into the high-dimensional implicit relationships between features. In addition, the embedding layer is designed with a multiheaded attention mechanism, which can mine the features that are most interesting to users during the model learning process and assign greater weights to these features to achieve interest diversification.
3. When conducting experiments on the public Zhihu dataset, the proposed model achieves satisfactory results in terms of accuracy and achieves an area under the curve (AUC) metric [15], that is, 3 percentage points higher than those of the existing models.

The remaining part of the article is organized as follows: In the second part, we present the research progress regarding the CTR prediction problem and analyze the advantages and shortcomings of the existing algorithms in detail. In the third part, we discuss some principles of the TAFM model in detail and introduce the algorithm implementation process. In the fourth part, we design experiments to compare and analyze the model proposed in this paper with the existing models. In the fifth part, we discuss the experimental results, draw conclusions, and provide an outlook on our future work.

## 2. Related Work

As mentioned earlier, previous works have produced acceptable results. Logistic regression and FMs, which are characterized by simplicity, speed, and low resource consumption, have been used in recommender systems for many years; however, these linear models lack complex interactions between features and have difficulty handling sparse features with high dimensionality. With deep learning making a splash in NLP and image processing, some recent works have also applied deep learning to CTR prediction tasks. The following section summarizes and discusses related work in terms of three aspects: deep learning, feature interaction, and attention mechanisms.

### 2.1. Deep Learning in CTR Prediction

As the volume of data increases, the business of CTR prediction is gradually becomes complex, machine learning models such as logistic regression cannot handle large-scale sparse matrices, and nonlinear models such as deep learning and neural networks models gradually become mainstream.

Li et al. [16] proposed a gated recurrent unit (GRU) model based on GRU neural networks to predict advertising CTRs, and by optimizing the improved step control method used by GRU neural networks, the model could reach the optimal point better and faster with fewer iterations. Jiang et al. [17] proposed a feature-based DNN (FDNN) model based on a fuzzy restricted Boltzmann machine (FRBM) and a Gaussian‒Bernoulli restricted Boltzmann machine (GBRBM). The FRBM and GBRBM, which automatically extract the hidden explanatory information from the original dataset, amplify the long-tailed important information and weaken the irrelevant information; thus, the prediction result of the FDNN is better than that of the LR model by 3.25%.

However, when applied to the CTR prediction task, a simple deep learning model does not consider the interactions of lower-order features and therefore, it cannot yield diverse feature interaction methods and does not tap into the diversity of user interests.

### 2.2. Feature Interaction in CTR Prediction

In addition to simply designing deep learning models for CTR prediction, more works have focused on the way in which features are crossed. To improve the feature interaction capability of a model and reduce its labor cost, the model structure is often used to automate the feature interaction process.

An FM [18] can map high-dimensional features to low-dimensional features and perform feature interaction via the inner product of low-dimensional feature vectors. A field-aware FM (FFM) [19] adds the concept of domain feature interaction to the FM feature interaction mechanism to further enhance the depth of feature interaction. A neural FM (NFM) [20] replaces the original FM algorithm with a biinteraction pooling layer by crossing feature vectors for feature intersection. Additionally, a DNN is added to modify the algorithm according to the relationships between the higher-order crossovers of features. An operation-aware neural network (ONN) [21] adds a second-order implicit crossover layer between its DNN and embedding layers to improve the resulting model. To reflect the weights of different feature crossovers, an attentional FM (AFM) [22] incorporates an attention mechanism to give different weights to the impacts of feature crossovers on CTR prediction and automatically learns such weights. An interaction-aware FM (IFM) [23] produced an improved model by incorporating both the output of the embedding layer and the original input into an implicit crossover operation; methods such as the adaptive factorization network (AFN) [24] yield improved models by adding a whole weight model on top of the original model. Other approaches further improve the ability of models to predict CTRs by modeling DNNs and FM in parallels; examples include DeepFM and wide and deep [25], which use the output of the embedding layer directly as the input of the FM and DNN. Some methods also extend FM feature crossover through the idea of deep learning, e.g., the deep and cross model [26], which was a follow-up to the wide and deep model; the associated paper proposed the DCN, which replaces the wide part with a cross implemented by a special network structure to automatically construct finite higher-order cross features and learn the corresponding weights. xDeepFM [27] utilizes a CIN network based on deep and cross with reference to the computation processes of convolutional neural networks (CNNs) and RNNs, adding pooling summation operations at each layer to improve the crossover network of deep and cross.

However, these models do not handle the embedding layer in a deep manner and do not consider multimodal features. For example, in CTR prediction realistic scenes may contain some important textual information.

### 2.3. Attention Mechanisms of CTR Prediction Models

With the great success of attention mechanisms in the field of NLP, the introduction of attention mechanisms to CTR prediction models has become a hot research topic.

As mentioned earlier, an AFM incorporates an attention mechanism into the improved FM model. Autoint [28] replaces the original attention layer with a multiheaded self-attention layer based on the AFM; the deep interest evolution network (DIEN) [29] and deep interest network (DIN) [30] replace the embedding layer with self-attention and multiheaded self-attention mechanism layers for model learning, respectively, and these two schemes have achieved good results in some complex scenarios with data clutter. The convolutional FM (CFM) [31] utilizes a self-attention mechanism to pool the output of the embedding layer and then uses a CNN to learn the relationships between features.

Similarly, these models do not consider multimodal user features, and they do not mine customer intent deeply enough. In summary, when designing a CTR prediction algorithm, not only should the solution to the feature sparsity problem be considered, but the low-order feature interactions and high-order feature interactions should also be considered. In addition, the mining of multimodal features also needs to be comprehensively considered. This is conducive to the accurate understanding of users' intentions and the mining of the user interest diversity.

## 3. Method

**Figure 1:**
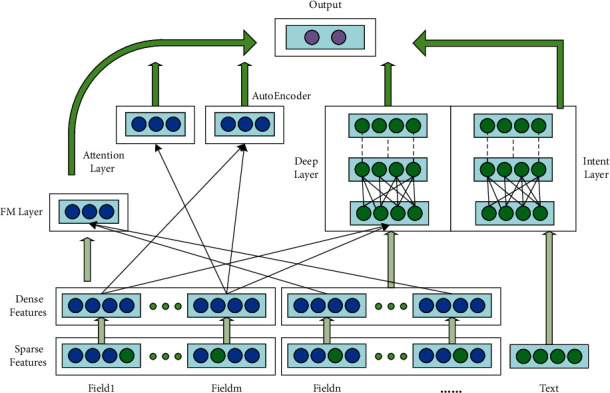
The overall pipeline of the proposed TAFM. The user attribute data go through the preprocessing layer and transform from sparse vectors into dense vectors. The dense vectors are computed in parallel in the FM, deep, intent understanding, attention, and autoencoder layers. Finally, the results of each layer are summed, sigmoid computation is performed, and the text data are fed directly to the intent mining layer in the deep layer.

In this section, we present the proposed TAFM in detail. The model aims to combine an attention mechanism and text understanding to achieve improved CTR prediction performance (see Figure 1). The TAFM in its entirety consists of a deep layer, an FM layer, an attention layer, and an autoencoder layer. The four model layers exist in a side-by-side manner, and the final output layer is a combination of the outputs of the four model layers. The deep layer consists of a DNN layer and an intent mining layer, which are used to process text data. These text data can include user comments or recommended text.

When user attribute data and text data are obtained, the user attribute data and items (which can be commodity attributes) are mapped to a common space. Notably, these user attribute data and item attribute data may be continuous or noncontinuous. Continuous data can be converted into categorical data by preprocessing. After performing data preprocessing, we design the attention layer and autoencoder layer to extract the high-dimensional features of the data and mine the associations between the features. The attention layer and autoencoder layer are connected in parallel with the deep layer and FM layer, which can reduce the model inference time. As mentioned earlier, textual data play a pivotal role in recommendation systems. Therefore, in the deep layer, we design a DNN layer and an intent mining layer. The inputs of the DNN layer include items such as user attributes and product attributes, and the inputs of the intent mining layer are text data.

As shown in Figure 1, the FM layer, attention layer, autoencoder layer and deep layer of the TAFM model share the embedding layer. The prediction result of the model can be written as in Equation (1):

(1) $$\begin{array}{c} {\overset{\wedge }{y}}_{TAFM}=\left(y_{FM}+y_{\text{Attention}}+y_{\text{AutoEncoder}}+y_{\text{Deep}}\right). \end{array}$$

where ${\overset{\wedge }{y}}_{TAFM}$ denotes the final prediction result of the TAFM model, *y*_*FM*_ denotes the calculation result of the FM layer, *y*_Attention_ denotes the prediction result of the attention layer, *y*_AutoEncoder_ denotes the calculation result of the autoencoder layer, and *y*_Deep_ denotes the calculation result of the deep layer.

### 3.1. Feature Embedding

The purpose of a recommendation system is to recommend items of interest to users, so it is necessary to model the attributes of users and items. The representations of user and item attribute directly affect the quality of the output recommendation results, so it is necessary to efficiently represent user interests and item attributes.

**Figure 2:**
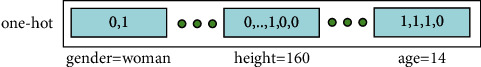
One-hot encoding representation of input features.

One-hot coding involves the representation of a categorical variable as a binary vector. A binary vector possesses only one activation point. In such a vector, only one feature value is not 0 (all others are 0). For example, a feature called “gender” has only two features, “male” and “female;” the feature value of “male” is coded as “10,” and the feature value of “female” is coded as “01.” The attributes of a user can be “gender = female, height = 160, age = 17,” where “gender” is a categorical feature and “age” and “height” are continuous features (see Figure 2).

**Figure 3:**
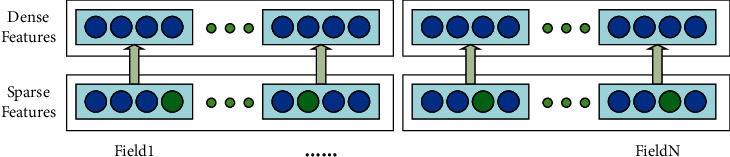
The embedding layer is represented by a sparse vector in a dense vector network.

Since a recommendation system has a large number of attributes, the feature dimensionality is high and sparse after one-hot encoding, so the embedding layer needs to map the high-dimensional sparse features into dense features from the input layer to the embedding layer (as shown in Figure 3). From Figure 3, the number of domain categories in the original dataset is *N*, and each domain *α* (1 ≤ *α* ≤ *n*) is represented as a low-dimensional vector *e*_*a*_ ∈ *R*^1×*k*^ after passing through the embedding layer, where *k* is the embedding dimensionality. The connection weight between the input layer and the embedding layer is *V*_*ij*_, indicating that the *i*-th feature is in the *j*-th dimension of the hidden vector after embedding. Therefore, the original feature input is converted into an embedding matrix *E*=(*e*_1_ ^*T*^, *e*_2_ ^*T*^, *e*_3_ ^*T*^,……,*e*_*n*_ ^*T*^)^*T*^, where *E* ∈ *R*^*n*×*k*^.

Features are generally classified into continuous features (also called numeric features) and categorical features (also called discontinuous features). We preprocess continuous-type features before inputting them into the embedding layer, as shown in Equation (2).

(2) $$\begin{array}{c} V_{\text{continuous}}=\left\{\begin{array}{cc} V_{\text{input}}-2, & \text{if }V_{\text{input}}\leq 2\text{,}\\ \log^{2}\left(V_{\text{input}}\right), & \text{if }V_{\text{input}}>2, \end{array}\right. \end{array}$$

where *V*_input_ denotes an original numerical feature and *V*_continuous_ denotes a preprocessed continuous feature. The purpose of doing this is to narrow down the original continuous-type features to a suitable range in which the continuous-type features are not too sparse.

### 3.2. FM Layer

One of the problems faced by recommendation systems is data sparsity, which can lead to poor training for algorithmic models, which in turn affects the final results of these recommendation systems. An FM model is based on linear regression and the introduction of a feature crossover term; the expression of an FM is shown in Equation (3):

(3) $$\begin{array}{c} y_{FM}=w_{0}+\sum_{i=1}^{n}w_{i}x_{i}+\sum_{i=1}^{n-1}\sum_{j=i+1}^{n}w_{ij}x_{i}x_{j}. \end{array}$$

where *w*_0_ denotes the constant bias and *n* denotes the number of features in the given sample.

In a case with sparse data, it is highly probable that *x*_*i*_ or *x*_*j*_ is 0, so it is difficult to train to obtain *w*_*i*_ _*j*_. An auxiliary vector *v*_*i*_=(*v*_*i*1_, *v*_*i*2_,…, *v*_*ik*_) is introduced to represent the feature *x*_*i*_, as shown in Equation (4).

(4) $$\begin{array}{c} V={\left(\begin{array}{cccc} v_{11} & v_{12} & ... & v_{1k}\\ v_{21} & v_{22} & ... & v_{2k}\\ \vdots & \vdots & \vdots & \vdots \\ v_{n1} & v_{n2} & \cdots & v_{nk} \end{array}\right)}_{n\times k}=\left(\begin{array}{c} v_{1}\\ v_{2}\\ \vdots \\ v_{n} \end{array}\right). \end{array}$$

We solve for *w*_*ij*_ with *v*_*i*_ · *v*_*j*_, as expressed in Equation (5):

(5) $$\begin{array}{c} \hat{W}=VV^{T}=\left(\begin{array}{c} v_{1}\\ v_{2}\\ \vdots \\ v_{4} \end{array}\right)\left(\begin{array}{cccc} v_{1}{\;}^{T} & v_{2}{\;}^{T} & \cdots & v_{n}{\;}^{T} \end{array}\right). \end{array}$$

Therefore, The FM is calculated as shown in Equation (6). The specific calculation procedure is described in the literature [8]:

(6) $$\begin{array}{c} y_{FM}=\text{bias}+<{\text{bias}}_{i},x_{i}>+\sum_{j_{1}=1}^{d}\sum_{j_{2}=j_{1}+1}^{d}\langle e_{i,f_{j_{2}}},e_{j,f_{j_{1}}}\rangle x_{i}\cdot x_{j}. \end{array}$$

where bias_*i*_ ∈ *R*^*d*^, *e* ∈ *R*^*k*^ (d is the number of features and *e* is the vector dimension); 〈bias_*i*_, *x*〉_*i*_ denotes the first-order importance between features. 〈*e*_*i*,*f*_*j*_2___, *e*_*j*,*f*_*j*_1___〉 is the second order between feature interactions, where the vector feature *e* of each feature is implemented through the embedding layer.

### 3.3. Attention Layer

**Figure 4:**
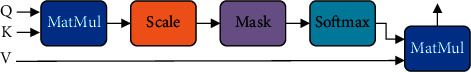
Scaled dot product structure diagram.

The TAFM is an improvement over the DeepFM model, which uses average pooling to obtain a consistent and nondiverse representation of user interests. To solve the problems of DeepFM, the TAFM introduces a multiheaded attention mechanism after embedding the user behavior list. With the introduction of the multihead attention mechanism, the model can specify the content that the global context should focus on and then provide more attention resources for this content. Each head in the multihead attention mechanism is computed in exactly the same way but with different parameters, thus enabling feature representation from multiple subspaces. This feature allows the recommender system to learn more useful information. The basis of the multihead attention mechanism is its scaling point attention mechanism, and the structure of this mechanism is shown in Figure 4.

The input of the scaling point attention mechanism [8] is composed of *Q*, *K*, and *V*. The scaled dot-product attention mechanism first computes the dot product of *Q* and *K*. After that, a scaling layer is added to suppress the growth rate of the result. The final output is shown in Equation (8):

(7) $$\begin{array}{c} \text{Attention}\left(Q,K,V\right)=\text{softmax}\left(\frac{QK^{T}}{\sqrt{d_{k}}}\right)V. \end{array}$$

where *d*_*k*_ is the dimension of the key. When *d*_*k*_ is large, the dot product result will become very discrete, so the dot product result is normalized by dividing it by $\sqrt{d_{k}}$. In the abovementioned equation, *Q* is the query matrix (multiple query vectors put together in the form of a matrix), *K*, and *V* are the keys and values represented by the matrix, respectively.

**Figure 5:**
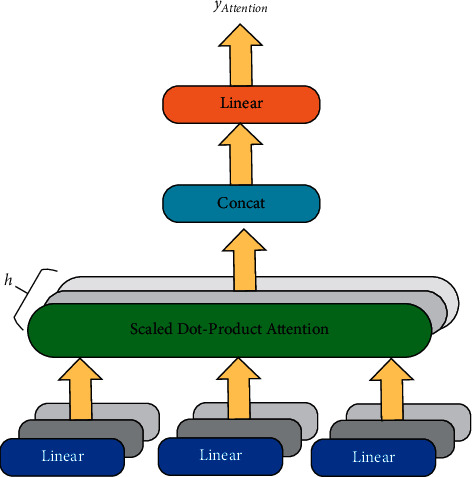
Scaled dot-product attention mechanism structure diagram.

The multiheaded attention mechanism [32] operates by performing *h* different linear mappings of *Q*, *K*, and *V* and scaling dot-product attention calculations on the *h* different mapping results in parallel. The results of the scaled dot-product attention process are then stitched and fed into the linear mapping layer (see Figure 5).

The output of the multihead attention mechanism is as shown in Equation (9):

(8) $$\begin{array}{c} y_{\text{Attention}}=\text{MultiHead}\left(Q,K,V\right)=\text{Concat}\left({\text{head}}_{1},\ldots ,{\text{head}}_{h}\right)W^{O},\\ {\text{head}}_{i}=\text{Attention}\left(QW_{i}{\;}^{Q},KW_{i}{\;}^{K},VW_{i}{\;}^{V}\right). \end{array}$$

where we assume that the vector dimensions of keys (i.e., queries) and values are *d*_*k*_ and *d*_*v*_, respectively, Then *W*_*i*_ ^*Q*^, *W*_*i*_ ^*K*^, *W*_*i*_ ^*V*^, *W*^*O*^ are linear transformation parameters in the abovementioned equation; *i* *=* 1, 2,…, *n*. *h* is the “head number” of multihead attention, the number of “heads” (*h* = 8 in the model).

### 3.4. Autoencoder Layer

After the previous discussion, it is clear that an important factor affecting the recommendation system is the features extracted by the model. A traditional linear model only considers independent individual features, while the FM algorithm considers the interactions of second-order features. To retain more information in the high-dimensional space and further tap into the high-dimensional implicit relationships between features, it is necessary to further improve the existing recommendation algorithm at the feature extraction level.

In view of the advantages of autoencoder networks with respect to feature learning, we design a convolutional autoencoder [33]. A convolutional autoencoder is an unsupervised deep learning network model that reconstructs the input data through the network for effective coding. The number of nodes in the middle layer of the convolutional autoencoder network is smaller than the number of nodes on both sides, which allows for effective feature learning and dimensionality reduction.

**Figure 6:**
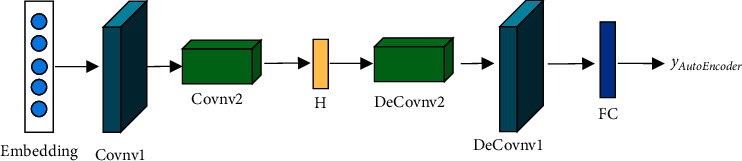
Structure diagram of the convolutional autoencoder.

As shown in Figure 6, after the original features are processed by the embedding layer, feature extraction is performed with the convolutional autoencoder. The convolutional autoencoder contains two encoder layers (i.e., Covnv1 and Covnv2), an intermediate layer *H,* and two decoder layers (DeCovnv2 and DeCovnv1), which are finally processed by a fully connected layer.

### 3.5. Deep Layer

In addition to static attributes, such as user characteristics and item characteristics, textual information is also an important factor that affects a recommendation system.

To fully exploit the implicit information contained in the given text, we embed the text attributes into the deep layer of the TAFM. First, we design a DNN layer for item feature extraction. Second, to deeply mine the semantic information of the input text sequences, we design an intent mining layer. The text component in the intent mining layer can capture the sequence information and semantic information in the text, and the text attention component in the intent mining layer can capture the most important words and semantic information in the sentences without using additional knowledge or an NLP system. In addition, the N-gram text feature extraction component in the intent mining layer obtains an N-gram feature representation of the input sentence via one-dimensional convolution.

**Figure 7:**
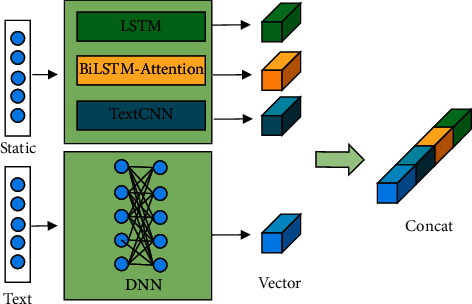
The structure of the deep feature extraction layer.

In summary, the DNN layer in the deep layer extracts static user attribute features. The text information is extracted by the text component, the text attention component, and the N-gram text feature extraction component in the deep layer. Finally, the vectors output by the DNN layer and the intent mining layer are stitched together to form the output of the deep layer (see Figure 7). In the figure, “static” refers to static attributes, such as the user's age, height, and other attributes. “Text” refers to the text attributes. Text attributes are dynamic attributes in the recommendation system. The diversity of text is also the source of the diversity of user interests. We recommend different texts to the same user to tap into the different interests of this user. The following section introduces the designed components.

#### 3.5.1. DNN Component

This component consists of three linear layers and three activation function layers arranged in a sequential crossover. The activation function uses the rectified linear unit (ReLU) function in Equation (10):

(9) $$\begin{array}{c} f_{RELU}=\max\left(0,x\right). \end{array}$$

The original features of the recommender system have the problem of data sparsity, and the ReLU function can effectively load the model to a lesser extent, so the ReLU function is suitable here.

#### 3.5.2. Text Component

**Figure 8:**
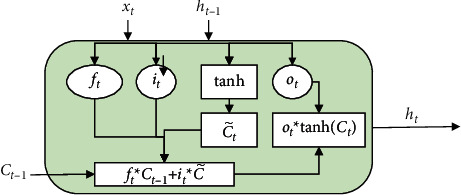
Single neuron structure of LSTM.

An RNN can effectively solve sequence problems. In the past few years, RNNs have achieved great success in speech recognition, language modeling, text translation, and other tasks, but an RNN has the problems of gradient explosion and gradient disappearance due to the presence of continuous cyclic input information. The text component LSTM [34] introduces a gating unit, which can solve the abovementioned problems faced by RNNs (see Figure 8). The mathematical expression of the text component LSTM is shown in Equation (11):

(10) $$\begin{array}{c} f_{t}=\sigma \left(W_{f}\cdot \left[h_{t-1},x_{t}\right]+b_{f}\right),\\ i_{t}=\sigma \left(W_{i}\cdot \left[h_{t-1},x_{t}\right]+b_{i}\right),\\ {\tilde{C}}_{t}=\tanh\left(W_{C}\cdot \left[h_{t-1},x_{t}\right]+b_{C}\right),\\ C_{t}=f_{t}*C_{t-1}+i_{t}*{\tilde{C}}_{t},\\ o_{t}=\sigma \left(W_{o}\cdot \left[h_{t-1},x_{t}\right]+b_{o}\right),\\ h_{t}=o_{t}*\;\tanh\left(C_{t}\right). \end{array}$$

where *x*_*t*_ represents the current input value, *h*_*t*_ represents the output of the current hidden layer, and *h*_*t*−1_ represents the output of the hidden layer at the previous moment. *C*_*t*_ represents the memory cell in the current hidden layer, *C*_*t*−1_ represents the memory cell in the hidden layer at the previous moment and ${\tilde{C}}_{t}$ represents the state of the memory cell. The function of the memory cell is to control the transmission of information in the memory unit. *i*_*t*_ represents the input gate, which is used to control the information that needs to be retained. *o*_*t*_ represents the output gate, which is used to control the information that must be output. *f*_*t*_ is the forget gate, used to control the information that needs to be discarded. *W*_*f*_, *W*_*i*_, *W*_*C*_, and *W*_*o*_ are weight matrices. *b*_*f*_, *b*_*i*_, *b*_*C*_, and *b*_*o*_ are bias matrices. *σ* is the sigmoid function. Both *sigmoid* and tanh are activation functions, and their expressions are shown in Equation (12):

(11) $$\begin{array}{c} \sigma \left(x\right)=\frac{1}{1+e^{-x}},\\ \tanh\left(x\right)=\frac{e^{x}-e^{-x}}{e^{x}+e^{-x}}. \end{array}$$

#### 3.5.3. Text Attention Component

To dig deeper into the semantic information of keywords, we design the text attention component. This component is based on the neural attention mechanism so that it can capture the most important semantic information in sentences, and the attention mechanism can automatically focus on words with decisive impacts.

Each training sequence of the BiLSTM consists of forwarding and backward LSTM layers, and the input *x* at time *t* in the BiLSTM is extracted by the BiLSTM layer features so that the model can learn the text sequence relationships in a more comprehensive manner. The BiLSTM can be viewed as two unidirectional LSTM units, so the hidden layer state of the BiLSTM at time *t* is obtained by weighting the forward hidden layer state *h*_1_, and the backward hidden layer state *h*_2_ is weighted and summed as in Equation (13):

(12) $$\begin{array}{c} \overset{⟶}{h_{t}}=LSTM\left(x_{t},{\overset{⟶}{h}}_{t-1}\right),\\ \overset{\leftarrow }{h_{t}}=LSTM\left(x_{t},{\overset{\leftarrow }{h}}_{t-1}\right),\\ h_{t}=w_{t}\overset{⟶}{h_{t}}+v_{t}\overset{\leftarrow }{h_{t}}+b_{t}. \end{array}$$

where *w*_*t*_ and *v*_*t*_ represent the weights corresponding to $\overset{⟶}{h_{t}}$ and $\overset{\leftarrow }{h_{t}}$ at time *t*, respectively, and *b*_*t*_ represents the bias corresponding to the hidden layer state at time *t*.

The input of the attention mechanism layer is the output vector *h*_*t*_ processed by the BiLSTM neural network layer in the previous layer. At this moment, *h*_*t*_ is the new *h*_*t*_ obtained by the weighted summation of the forward hidden layer state $\overset{⟶}{h_{t}}$ and the backward hidden layer state $\overset{\leftarrow }{h_{t}}$. The specific formula of the attention mechanism layer is shown in Equation (14):

(13) $$\begin{array}{c} u_{t}=\tanh\left(w_{w}h_{t}+b_{w}\right),\\ a_{t}=\frac{\exp\left(u_{t}{\;}^{T}u_{w}\right)}{\sum_{t}\exp\left(u_{t}{\;}^{T}u_{w}\right)},\\ s_{t}=\sum_{t}a_{t}h_{t}, \end{array}$$

where *h*_*t*_ is the output vector processed by the BiLSTM neural network layer in the previous layer, *w*_*w*_ is the weight coefficient, and *b*_*w*_ is the bias coefficient. *u*_*t*_ represents the energy value determined by *h*_*t*_, *a*_*t*_ is the weight coefficient of each hidden layer state in the new hidden layer, *u*_*w*_ is an attention matrix, that is, randomly initialized and continuously updated during the training process, and *s*_*t*_ is the output vector of the attention mechanism. The softmax function is used to perform corresponding calculations on the output layer, and the formula is as follows:

(14) $$\begin{array}{c} y_{j}=\text{softmax}\left(w_{j}s_{t}+b_{j}\right), \end{array}$$

where *w*_*j*_ denotes the weight coefficient matrix to be trained from the attention mechanism layer to the output layer, *b*_*j*_ denotes the bias corresponding to the training process to be performed, and *y*_*j*_ is the output vector.

**Figure 9:**
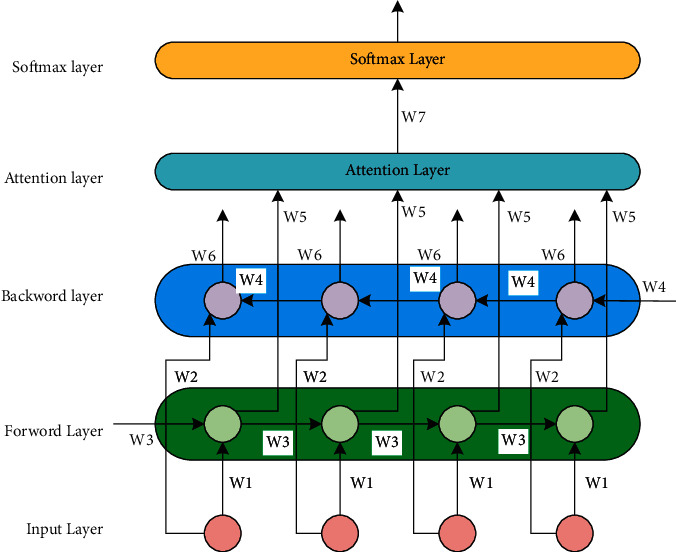
BiLSTM-Attention feature extraction component.

The text attention component can fully mine not only the semantic information of text but also the keyword information of text (see Figure 9).

#### 3.5.4. N-Gram Feature Extraction Component

**Figure 10:**
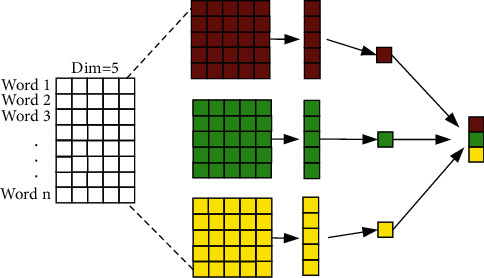
N-gram feature extraction component.

The TAFM designs an N-gram [35] text feature extraction component to obtain the N-gram feature representation of a sentence via one-dimensional convolution (see Figure 10). In the recommendation system, the input text is composed of several consecutive words, so the height of the convolution kernel can be set to extract the correlations of adjacent words in the user's behavior sequence; this approach not only considers the meaning of words in dynamic behavior text but also takes the word order and context into account. In Figure 10, the height of the convolution kernel is set to 5 to extract 5-gram features from the text. After the convolutional layer, the data are passed through the ReLU activation function, the maximum pooling layer, and the fully connected layer to obtain the text features. The advantage of using convolutional kernels over the N-gram model in traditional machine learning is that only the composition of consecutive words is considered, and the word list in the training set does not grow explosively.

## 4. Experiments and Results

In this section, we conduct experiments and analyze the results. First, we introduce the hardware environment and dataset of our experiments, and second, we describe some metrics used to evaluate the experimental results in detail. Finally, we conduct a comparison experiment and discuss our experimental results.

### 4.1. Experimental Environment and Dataset

Our experimental code uses the PyTorch-1.7 framework and the underlying Python3 language. The operating system used for the experiments is the Centos7 operating system. The server used for the experiments is a Nvidia GeForce RTX with 12 GB of video memory and CUDA version 10.1.

### 4.2. Dataset

Zhihu (https://www.zhihu.com/), the Chinese Internet's high-quality question and answer community and an original content platform for creators, was officially launched in January 2011 with the brand mission of “allowing people to better share their knowledge, experience and insights and find their own answers.” In such knowledge sharing or Q&A communities, the number of questions far exceeds the number of quality responses. Therefore, how to connect knowledge, experts and users and increase the willingness of experts to answer questions become the central issues faced by such services. This paper uses Zhihu's public dataset, which is located at https://www.biendata.xyz/competition/zhihu2019/data/. The dataset includes Zhihu's question information, user profiles, user answer records, user invitation acceptances, etc. It mainly includes the following contents.

1. Issue information. Includes <question ID, question creation time, question topic, question text, question description>, etc.
2. The user's answers. Include <answer ID, question ID, author ID, text of answer, answer time, number of likes, number of favorites, number of thanks, number of comments>, etc.
3. User persona data. Include <user ID, gender, active frequency, following topics, long-term interests, salt value>, etc.
4. Word embedding vector. Includes <topic, token (word), single-word 64-dimensional embedding> and other data.
5. The most recent January invitation data. Include <question ID, user ID, invitation time, and whether answered>.

We execute a linkage operation on multiple data files based on the user IDs and question IDs. Finally, we collate 16 categorical features and 7 numerical features for model training (see Table 1).

During the categorical feature preprocessing step, we perform hard coding, i.e., direct coding with numbers, and we set null values to −1. For continuous numerical feature processing, we perform normalization: after centering the data by the minimum value and then scaling them by the extreme difference (maximum–minimum), the data are shifted by the minimum value unit and converge to a value between [0, 1].

On the Zhihu platform, after a user asks a question, Zhihu recommends the question to other users who are suitable to answer it. Therefore, we recommend the question title and question-related attributes to users according to the requirements of the given business scenario. Here the question title is given as text data in the recommendation system.

### 4.3. Evaluation Metrics

In this paper, various evaluation criteria are used to test the model effects. The loss function and evaluation metrics used by the TAFM algorithm are described in the following section.

The cross entropy and root mean square error (RMSE) [36] are used as loss functions to evaluate the error value of the recommendation model with respect to the algorithm calculation, and the formulas for calculating the cross entropy and RMSE are shown in equation (16).

(15) $$\begin{array}{c} \text{Log}\_\text{Loss}=-\left[y\;\ln\bar{y}+\left(1-y\right)\ln\left(1-\bar{y}\right)\right],\\ \text{Log}\_\text{RMSE}=\frac{1}{N}\sum_{i=1}^{N}\left(y_{i}-\bar{y_{i}}\right), \end{array}$$

where *y* denotes the predicted result and $\bar{y}$ denotes the true value, *N* denotes the number of samples.

Precision and recall are calculated by confusion matrix [37], which can be used to evaluate how good or bad the recommendation model is in terms of recommendation effectiveness. The parameters and calculation methods involved are as follows: a true positive (*TP*) means that a positive class is predicted as positive; a true negative (*TN*) means that a negative class is predicted as negative; a false positive (*FP*) means that a negative class is predicted as positive; and a false negative (*FN*) means that a positive class is predicted as negative. The formulas for the recall and precision metrics are shown in equation (17).

(16) $$\begin{array}{c} \text{recall}=\frac{TP}{TP+FN},\\ \text{precision}=\frac{TP}{TP+FP}. \end{array}$$

However, both recall and accuracy are focused metrics and cannot be combined. Therefore, we combine the accuracy (ACC) and *F*1-score [38] values to determine the effectiveness of the model, as shown in equation (18).

(17) $$\begin{array}{c} Acc=\frac{TP+TN}{TP+TN+FP+FN},\\ F1\_\text{Score}=\frac{2\times \text{precision}\times \text{recall}}{\text{precision}+\text{recall}}. \end{array}$$

The AUC [39] evaluates the merit of the obtained recommendation results from the perspective of sample probability, which is simply introduced as the probability of acquiring a positive sample, that is, greater than the negative sample in a pair of randomly selected samples (one positive sample and one negative sample); the model obtained from training is used to predict these two samples, as shown in Equation (18).

(18) $$\begin{array}{c} AUC=\frac{\sum I\left(P_{\text{positive}},P_{\text{negative}}\right)}{M\times N},\\ I\left(P_{\text{positive}},P_{\text{negative}}\right)=\left\{\begin{array}{cc} 1, & P_{\text{positive}}>P_{\text{negative}},\\ 0.5, & P_{\text{positive}}=P_{\text{negative}},\\ 0, & P_{\text{positive}}>P_{\text{negative}}, \end{array}\right. \end{array}$$

where *M* denotes the number of positive samples, *N* denotes the number of negative samples, and *M* × *N* represents the total number of sample pairs, *P*_positive_ refers to the probability of getting a positive sample for the prediction, *P*_negative_ refers to the probability of getting a negative sample for the prediction.

### 4.4. Experimental Hyperparameter Settings

The inputs of the TAFM include the files related to the user features, question features, and user question cross features of the Zhihu dataset. The preprocessing and input procedures of this process are as follows:

(1) The user information mainly includes the user ID, gender, active frequency, topics followed, long-term interests, and salt value. The question information includes the question ID, question creation time, question topic, question text, and question description. The cross features of a user question mainly include the user's answer and the user's invitation. The specific fields of the user's answer are the answer ID, question ID, author ID, text of answer, answer time, number of likes, number of favorites, number of thanks, and number of comments; the specific fields of the user's invitation are the question ID, user ID, invitation time, and whether to answer. These attributes are similar to the internal connections in the MySQL database, and the key to these connections is mainly the ID of each attribute. The text attribute is selected as the title of the text and mapped to a 64-dimensional word vector.
(2) The attributes of the data are processed. Attributes are divided into continuous and discrete attributes, as introduced in Table 1. The continuous and discrete variables are embedded to obtain a dense vector. In the model, we call the features that have not been processed by embedding the original features, and the features that have been processed by embedding are called the embedded features. Therefore, three kinds of features are utilized in our algorithm: original features, embedded features (original features with dense embedding-based processing), and text. In the deep layer of the algorithm, we input the text into the intent mining layer and the embedded features into the DNN layer. In the FM layer, attention layer, and autoencoder layer, we use only the embedding features as inputs.
The data dimensions are summarized as follows: the original dimensionality of the input data is 23, and the dimensionality after performing embedding-based processing [16, 23]. The input of the depth layer requires the embedded data dimensionality to be large, and the dimensionality is 23 times 16, totaling 368 dimensions.
(3) The deep layer passes the input text through the LSTM, BiLSTM-Attention, and CNN layers, and the obtained output is stitched with the input of the deep layer, after which it is processed into a one-dimensional vector by full concatenation.
    ① The main parameters of the LSTM model are as follows: the input dimensionality is 64, the hidden layer dimensionality is 10, and the number of network layers is 4.
    ② In BiLSTM-Attention, the last layer is the fully connected layer, the input dimensionality of the fully connected layer is two times that of the hidden layer, and the output dimensionality is 10.
     ③ In CNN, the number of input channels for the convolutional layer is 1, the number of output channels is 3, the height of the convolutional kernel is 5, and the width of the convolutional kernel is 64. The convolutional layer is followed by a ReLU function and a maximum pooling layer. The pooling layer has a height of 5 and a height of 1. The fully connected layer of the CNN layer has an input dimensionality of 21 and an output dimensionality of 10.

Overall, the deep layer input vector has 368 dimensions, and the LSTM, BiLSTM-Attention, and CNN outputs all have 10 dimensions. The four output vectors are spliced, and the input of the fully connected layer has 1 dimension. We design a weight_decay parameter in the model optimizer to prevent the model from overfitting, and we design a dropout mechanism inside the model.

### 4.5. Experimental Results

This experiment utilizes 9,489,162 data points. We partition the data according to an 8 : 1 : 1 ratio, and after the partitioning process is completed, the data are used as the training set, validation set, and test set. In this paper, the AFM, the CCPM, the DCN, DeepFM, Factorization Machine supported Neural Network (FNN) [40], multiple linear regression (MLR) [41], the NFM, Product-based Neural Network (PNN) [42], wDeepFM, and xDeepFM are selected as the baseline models, and the models are graphically compared below.

**Figure 11:**
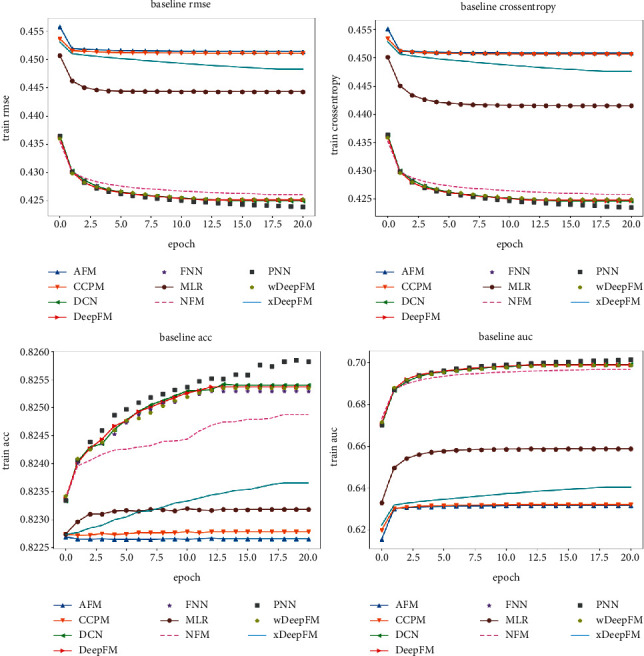
Performance of the baseline models on the training set.

The performance of the baseline models on the training set during training is measured with the RMSE loss, cross-entropy loss, accuracy, and AUC score metrics, which are presented in the four subplots (see Figure 11). The PNN model, DCN model, DeepFM model, xDeepFM model, and wDeepFM model produce the fastest-decreasing loss values during training. The AFM and CCPM differ slightly in terms of their loss functions, and the cross-entropy loss values converge faster; this is due to the RMSE loss function, which is a measure of the distances between feature maps. The goal is to extract feature map inferences with consistency, while the cross-entropy loss is a measure of the similarity between two probability distributions *p* and *q*. In feature engineering, this loss is used to measure the importance of variables, so cross-entropy is often used for classification purposes. In terms of accuracy metrics, the accuracy of each model fluctuates widely but is relatively smooth in the corresponding AUC curve.

**Figure 12:**
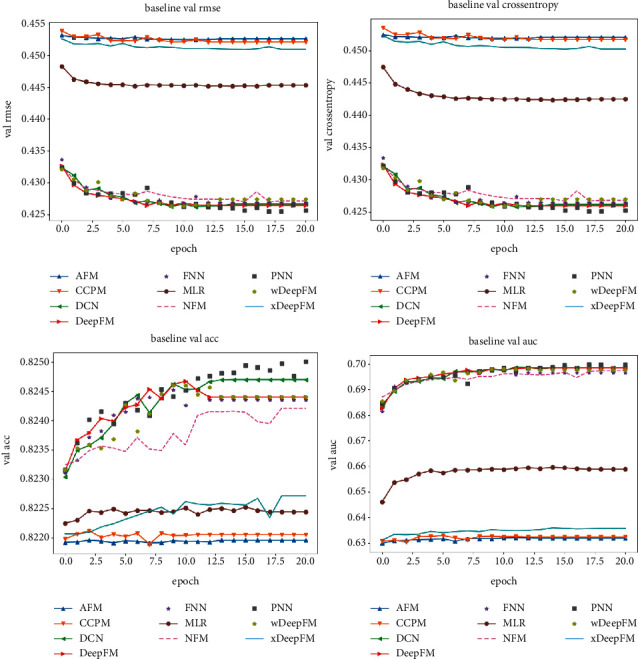
Performance of the baseline models on the validation set.

Then, we conduct a comparison of the validation set involving the baseline models (see Figure 12). As shown in the figure, the MLR model is most likely to reach a minimal cross-entropy loss value, but its RMSE does not exhibit a consistent decrease. In terms of the ACC metrics, the best results of our model are achieved by the 10th round of model training, followed by those of DeepFM and xDeepFM. After more than 10 rounds, the model accuracy fluctuates, and the PNN model accuracy continuously fluctuates. The AUC score of the PNN is unstable, but its index performance is excellent because the PNN model explicitly carries out feature interaction and fails to adequately learn some hidden layer features. In addition, the PNN carries out feature interaction via the outer product operation, which is computationally intensive. DeepFM and xDeepFM do not exhibit large fluctuations; this is because their features are deep after embedding, so their AUC curves are smoother and perform better.

Based on the abovementioned data performance, to enable our recommendation model to incorporate text, we design the TAFM recommendation algorithm based on DeepFM. To fully explore the different importance levels of various cross features, the TAFM model is designed with a self-attention mechanism. Additionally, the TAFM is designed with a convolutional self-encoder to process the embedded features so that more nonlinear hidden layer features can be mined. In the deep layer, the text component, text attention component, and N-gram text feature extraction component are designed to separately process the input text (see Table 2).

As seen in Table 2, when the learning rate is set to 0.01 and the batch size is set to 1024, the TAFM model achieves optimal performance, with an accuracy of 0.8269 and an AUC score of 0.73018.

Combining the evaluation metrics, we conduct experiments on the baseline models and the TAFM on the test set (see Table 3).

As seen in Table 3, the TAFM accuracy is higher than that of the existing baseline models, and the AUC score is at least 3 percentage points higher than that of the existing models. The TAFM model learns the features of the given text, which makes the recommendation results more accurate. Second, the outermost self-attention layer of the model can learn the importance of different cross features, and the self-roller encoding layer can learn the invisible features contained in the text features.

Our analysis of each model is compared as follows: AFM adds the expressiveness of attention, which increases the interpretability of the model to some extent. But it is still a shallow model, and the model does not learn the higher-order cross-sectional features. Therefore, AFM performs the worst in terms of AUC score. One of the difficulties of CCPM using CNN for feature extraction in the CTR task is that it computes local feature combinations and cannot effectively capture global combined features; similarly, the AUC score of CCPM model is about 0.63. DCN has the capability of explicit higher-order feature crossover, which can transfer the original information in CrossNet, but DCN is bitwise in performing crossover, and the final DCN output has some limitations, and this form limits the expressiveness of the model to some extent. The DeepFM model can learn both low-order and high-order features, sharing the information representation of features, but the DNN is still implicit for learning high-order features. The FNN offline training FM obtains embedding, that is, input to the NN, which is equivalent to introducing a priori expert experience, accelerating the training and convergence of the model, while the NN model eliminates the learning feature embedding step and has low training overhead. However, FNN is not an end-to-end two-stage model, which is not conducive to online learning. The pretraining embedding is limited by the FM model, and only the high-order crossover of features is considered in the FNN, and the low-order feature information is not retained. So the AUC scores of the DCN, DeepFM, and FNN models are approximately 0.698. The MLR model is a generalization of the linear LR model, and although the MLP can theoretically approximate any function with arbitrary accuracy, the more generalized the representation, the less easy it is to fit a specific pattern of specific data. The AUC score of the model is approximately 0.658. NFM uses the bi-interaction layer (bi-linear interaction) structure for second-order cross information so that the information of cross features can be better learned by the DNN structure and reduce the difficulty of DNN in learning higher-order cross feature information. An obvious drawback is that it does not consider any interaction between features. Therefore, these deep learning methods must rely entirely on the subsequent deep layer to learn meaningful feature interactions. The PNN network adds a product layer between the embedding layer and the fully connected layer to complete the feature combination. PNN and FNN are similar to other existing deep learning models in that it is difficult to extract low-order feature combinations efficiently. xDeepFM can learn both explicit higher-order feature crossover (CIN) and implicit higher-order feature crossover (DNN), and the CIN uses vectorwise crossover (instead of bitwise crossover in the DCN) in learning crossover features. However, the time complexity of xDeepFM is too high in practical computation, and the sum-pooling operation of the model will lose some information. wDeepFM learns both low-order feature crossover (wide part) and high-order feature crossover (deep part). However, the crossover features still need to be designed manually. The AUC scores of the abovementioned models range from 0.63 to 0.69. Our model TAFM is higher than the existing models in terms of accuracy and AUC metrics.

### 4.6. Ablation Study

To verify the validity of our model, we designed ablation experiments for the variants of the TAFM model. Among them, the design of all model variants is shown in Table 4.

The experimental results of the variant of the TAFM model are shown in Table 5. As seen in Table 5, when the TAFM model is stripped of the convolutional autoencoder and the multiheaded attention mechanism, the accuracy of the model is about 0.824 and the AUC score is about 0.697. The experimental results show that the convolutional autoencoder is designed so that more information in the high-dimensional space can be retained and the high-dimensional implicit relationships between features can be mined. In addition, the embedding layer is designed with a multiheaded attention mechanism, which can mine the features that are more interesting to users during the model learning process and assign more weights to these features to achieve interest diversification. When the TAFM model removes the text-related component, the model accuracy hovers around 0.825, and the AUC score is about 0.69. As seen in the experimental results, we design the BiLSTM-Attention for key information extraction of text. The textual attention component captures the most important semantic information in sentences using a bidirectional long and short-term memory network (BiLSTM) and an attention mechanism. The attention mechanism automatically focuses on the words that have a decisive impact on classification and captures the most important semantic information in the sentences. The n-gram text feature extraction component mines the n-gram features in the text. In summary, all the components of the model have a positive impact on the experimental results.

## 5. Conclusions

To address the current problem that relevant deep learning recommendation algorithms cannot provide recommendations based on text, this paper designs a text recommendation algorithm based on an attention mechanism. In the embedding layer, we propose a method to process numerical features and prevent the gaps between features from exploding through the transformation of these numerical features. In the deep layer, we propose a text feature extraction method, design a text layer for text feature extraction to solve the long-time dependence of text and design a text attention layer to extract key text information and capture the most important semantic information contained in sentences. The attention mechanism can automatically focus on the words that have decisive impacts on the classification process and capture the most important semantic information in sentences. An N-gram text feature extraction layer is designed to obtain the N-gram feature representations of sentences via one-dimensional convolution, and multiple text feature extraction components further mine the hidden layer features from the text that are useful for the recommendation system. In the overall algorithm, the proposed multiheaded attention mechanism can clarify the content that the global context should focus on and then provide more attention resources for this content. Finally, convolutional autoencoders are designed to learn the invisible features of interaction features. After an experimental comparison and analysis, the TAFM algorithm improves the AUC metric by 3 percentage points over those of the other algorithms. However, the algorithm in this paper also has a limitation: its time complexity is higher than that of DeepFM due to the large computational volume of the model. This will be improved in our follow-up work.

There are three directions to be explored in the future. First, we will explore the association between individual features of users and items through an additional representation layer that should be able to represent the order of user-item pairs so that our recommendation algorithm is more interpretable. Secondly, we need to simplify the representation of the text, which will make our model more advantageous for industrial replication. We will try to further extract textual information by short text clustering [43], ensemble learning, DistilBERT [44], and try to optimize the time complexity [45]. Finally, we would like to incorporate external data (e.g., knowledge graph, speech, images) into CTR prediction to mine the intent behind users with richer features.

## Figures and Tables

**Table 1:** Dataset field descriptions.

Field name	Description	Numeric type
m_sex	User gender	Discrete
m_access_frequencies	Frequency of user access	Discrete
m_twoA∼m_twoE	Anonymous fields for dichotomous user features	Discrete
m_categoryA∼ m_categoryE	Anonymous fields for user classification features	Discrete
m_num_interest_topic	Number of topics of interest to users	Discrete
num_topic_attention_intersection	Intersection count of user-focused topics and question-bound topics	Discrete
q_num_topic_words	Number of topics bound to an issue	Discrete
num_topic_interest_intersection	Intersection count of topics of interest to users and question-bound topics	Discrete
m_salt_score	User salt score	Continuous
m_num_atten_topic	Number of topics followed by users	Continuous
q_num_title_chars_words	Question title word count	Continuous
q_num_desc_chars_words	Number of characters in the problem description	Continuous
q_num_desc_words	Number of words in the problem description	Continuous
q_num_title_words	Number of words in the question title	Continuous
days_to_invite	Number of days since the invitation was created for the issue	Continuous

**Table 2:** Effect of different learning rates and batch sizes on the model.

Learning rate	Batch size	Accuracy	AUC	Log_loss
0.0001	2048	0.825604	0.697758	0.4256
0.0005	2048	0.825103	0.697491	0.4263
0.001	16	0.821059	0.673495	0.4414
0.001	32	0.825450	0.681666	0.4342
0.001	64	0.824624	0.717330	0.4216
0.001	128	0.826032	0.724808	0.4166
0.001	256	0.826041	0.726161	0.4162
0.001	512	0.825287	0.695343	0.4265
0.001	1024	**0.826938**	**0.730182**	0.4144
0.001	2048	0.826891	0.728792	0.4141
0.005	2048	0.825827	0.697286	0.4257
0.01	2048	0.823770	0.682339	0.4326
0.1	2048	0.822422	0.5	17.7577

**Table 3:** Performance of different models in the test set.

Algorithm model	Model accuracy	AUC	Log_loss
AFM	0.82291	0.63090	0.4506
CCPM	0.82296	0.63170	0.4504
DCN	0.82564	0.69840	0.4245
DeepFM	0.82543	0.69830	0.4247
FNN	0.82554	0.69740	0.425
MLR	0.82336	0.65860	0.4412
NFM	0.82528	0.69670	0.4254
PNN	0.82585	0.69970	0.4239
xDeepFM	0.82352	0.63430	0.4491
wDeepFM	0.82560	0.69790	0.4248
TAFM	**0.82694**	**0.73018**	0.4144

**Table 4:** Results of ablation experiment.

Model variants	Explanation
TAFM_ae	TAFM_ae refers to the removal of the convolutional autoencoder from the TAFM model.
TAFM_a	TAFM_a refers to the removal of the outermost multiheaded attention mechanism based on the TAFM model.
TAFM_ae_a	TAFM_ae_a refers to the TAFM model with both the outermost multiheaded attention mechanism and the convolutional autoencoder removed.
TAFM_l	TAFM_l refers to the TAFM model with the LSTM removed.
TAFM_b	TAFM_b refers to the removal of BiLSTM_Attention from the TAFM model.
TAFM_c	TAFM_c refers to the TAFM model with the textCNN removed.
TAFM_l_b	TAFM_l_b refers to the TAFM model with both LSTM and BiLSTM_Attention removed.
TAFM_l_c	TAFM_l_c refers to the base of TAFM model with both LSTM and TextCNN removed.
TAFM_b_c	TAFM_b_c refers to the removal of both BiLSTM_Attention and TextCNN on top of the TAFM model.
TAFM_b_l_c	TAFM_b_l_c refers to the TAFM model with BiLSTM_Attention, LSTM and TextCNN removed at the same time; in other words, this variant removes the entire text module.

**Table 5:** Results of ablation experiment.

Algorithm model	Model accuracy	AUC	Log_loss
TAFM_ae	0.82497	0.69691	0.4266
TAFM_a	0.82490	0.69709	0.4265
TAFM_ae_a	0.82485	0.69743	0.4264
TAFM_l	0.82478	0.69659	0.4272
TAFM_b	0.82573	0.69546	0.4262
TAFM_c	0.82515	0.69596	0.4265
TAFM_l_b	0.82543	0.69676	0.4269
TAFM_l_c	0.82516	0.69707	0.4266
TAFM_b_c	0.82607	0.69815	0.4251
TAFM_b_l_c	0.82051	0.69554	0.4268
TAFM	**0.82694**	**0.73018**	0.4144

## Data Availability

The data used to support the findings of this study are available at https://www.biendata.xyz/competition/zhihu2019/data/.

## References

[1] Warren J. (2015). *Big Data: Principles and Best Practices of Scalable Realtime Data Systems*.

[2] Ricci F., Rokach L., Shapira B. (2011). Introduction to recommender systems handbook. *Recommender Systems Handbook*.

[3] Cervantes J., Garcia-Lamont F., Rodríguez-Mazahua L., Lopez A. (2020). A comprehensive survey on support vector machine classification: applications, challenges and trends. *Neurocomputing*.

[4] Wagena M. B., Goering D., Collick A. S. (2020). Comparison of short-term streamflow forecasting using stochastic time series, neural networks, process-based, and bayesian models. *Environmental Modelling & Software*.

[5] Hassanipour S., Ghaem H., Arab-Zozani M. (2019). Comparison of artificial neural network and logistic regression models for prediction of outcomes in trauma patients: a systematic review and meta-analysis. *Injury*.

[6] Zhang Y., Dai H., Xu C. Sequential click prediction for sponsored search with recurrent neural networks.

[7] Xu J., Hu Z., Zou J. (2021). Personalized product recommendation method for analyzing user behavior using DeepFM. *Journal of Information Processing Systems*.

[8] Rendle S. Factorization machines.

[9] Chen Q., Yu S., Guo Z., Jia Y. Estimating ads’ click through rate with recurrent neural network.

[10] Wang R., Fu B., Fu G., Wang M. Deep & cross network for ad click predictions.

[11] Lindemann B., Maschler B., Sahlab N., Weyrich M. (2021). A survey on anomaly detection for technical systems using LSTM networks. *Computers in Industry*.

[12] Lu W., Li J., Wang J., Qin L. (2021). A CNN-BiLSTM-AM method for stock price prediction. *Neural Computing & Applications*.

[13] Tian J., Yu J., Weng C., Zou Y., Yu D. (2022). Improving Mandarin end-to-end speech recognition with word N-gram language model. *IEEE Signal Processing Letters*.

[14] Kattenborn T., Leitloff J., Schiefer F., Hinz S. (2021). Review on convolutional neural networks (CNN) in vegetation remote sensing. *ISPRS Journal of Photogrammetry and Remote Sensing*.

[15] Muschelli J. (2020). ROC and AUC with A binary predictor: a potentially misleading metric. *Journal of Classification*.

[16] Li H., Duan H., Zheng Y., Wang Q., Wang Yu (2020). A CTR prediction model based on user interest via attention mechanism. *Applied Intelligence*.

[17] Zhang W., Zhang X., Wang H. (2019). High-order factorization machine based on cross weights network for recommendation. *IEEE Access*.

[18] Anh-Phuong T. Factorization machines with follow-the-regularized-leader for CTR prediction in display advertising.

[19] Juan Y., Zhuang Y., Chin W., Lin C.-J. Field-aware factorization machines for CTR prediction.

[20] He X., Chua T.-S. Neural factorization machines for sparse predictive analytics.

[21] Yang Y., Xu B., Shen S., Shen F., Zhao J. (2020). Operation-aware neural networks for user response prediction. *Neural Networks*.

[22] Xiao J., Ye H., He X., Zhang H., Wu F., Chua T.-S. Attentional factorization machines: learning the weight of feature interactions via attention networks.

[23] Hong F., Huang D., Chen Ge Interaction-aware factorization machines for recommender systems.

[24] Cheng W., Shen Y., Huang L. Adaptive factorization network: learning adaptive-order feature interactions.

[25] Cheng H.-T., Koc L., Harmsen J., Shaked T., Chandra T., Aradhye H. Wide & deep learning for recommender systems.

[26] Huang G., Chen Q., Deng C. (2020). A new click-through rates prediction model based on Deep&Cross network. *Algorithms*.

[27] Lian J., Zhou X., Zhang F., Chen Z., Xie X., Sun G. xDeepFM: combining explicit and implicit feature interactions for recommender systems.

[28] Song W., Shi C., Xiao Z., Duan Z., Xu Y., Zhang M. Autoint: automatic feature interaction learning via self-attentive neural networks.

[29] Zhou G., Mou Na, Fan Y. Deep interest evolution network for click-through rate prediction.

[30] Zhou G., Zhu X., Song C., Fan Y., Han Z., Xiao Ma Deep interest network for click-through rate prediction.

[31] Xin X., Chen Bo, He X., Wang D., Ding Y., Jose J. CFM: convolutional factorization machines for context-aware recommendation.

[32] Niu Z., Zhong G., Yu Y. (2021). A review on the attention mechanism of deep learning. *Neurocomputing*.

[33] Pang S., Gao L. (2022). Multihead attention mechanism guided convLSTM for pixel-level segmentation of ocean remote sensing image. *Multimedia Tools and Applications*.

[34] Akram M. W., Salman M., Bashir M. F., Salman M. S., Gadekallu T. R., Javed A. R. (2022). A novel deep auto-encoder based linguistics clustering model for social text. *Transactions on Asian And Low-Resource Language Information Processing*.

[35] Jalayer M., Orsenigo C., Vercellis C. (2021). Fault detection and diagnosis for rotating machinery: a model based on convolutional LSTM, fast fourier and continuous wavelet transforms. *Computers in Industry*.

[36] Liu Y., Wang Li, Shi T., Li J. (2022). Detection of spam reviews through A hierarchical attention architecture with N-gram CNN and Bi-LSTM. *Information Systems*.

[37] Chicco D., Warrens M. J., Jurman G. (2021). The coefficient of determination R-squared is more informative than SMAPE, MAE, MAPE, MSE and RMSE in regression analysis evaluation. *PeerJ Computer Science*.

[38] Markoulidakis I., Rallis I., Georgoulas I., Kopsiaftis G., Doulamis A., Doulamis N. (2021). Multiclass confusion matrix reduction method and its application on net promoter score classification problem. *Technologies*.

[39] DeVries Z., Locke E., Hoda M., Moravek D., Phan K., StrattonKingwellWaiPhan P. (2021). Using A national surgical database to predict complications following posterior lumbar surgery and comparing the area under the curve and F1-score for the assessment of prognostic capability. *The Spine Journal*.

[40] Yuan Z., Yan Y., Sonka M., Yang T. Large-scale robust deep auc maximization: a new surrogate loss and empirical studies on medical image classification.

[41] Zhou F., Zhou H., Yang Z., Yang L. (2019). EMD2FNN: a strategy combining empirical mode decomposition and factorization machine based neural network for stock market trend prediction. *Expert Systems with Applications*.

[42] Shams S. R., Jahani A., Kalantary S., Moeinaddini M., Khorasani N. (2021). The evaluation on artificial neural networks (ANN) and multiple linear regressions (MLR) models for predicting SO2 concentration. *Urban Climate*.

[43] Qu Y., Fang B., Zhang W. (2019). Product-based neural networks for user response prediction over multi-field categorical data. *ACM Transactions on Information Systems*.

[44] Akram M. W., Salman M., Bashir M. F., Salman S. M. S., Gadekallu T. R., Javed A. R. (2022). A novel deep auto-encoder based linguistics clustering model for social text. *Transactions on Asian and Low-Resource Language Information Processing*.

[45] Iqbal A. R., Jalil F., Gadekallu Z., Kryvinska T. R., Kryvinska N. (2022). Authorship identification using ensemble learning. *Scientific Reports*.

